# Emerging Role of BTK Inhibitors in Multiple Sclerosis: From Immunobiology to Clinical Translation

**DOI:** 10.3390/brainsci16060634

**Published:** 2026-06-12

**Authors:** Aashray Raj, Vansh Patel, Mehak Dang, Aken Kayastha, Yusuf Kagzi, Praveen Nandha Kumar Pitchan Velammal, Nidhi Agrawal, Kushagra Sharma, Nicholas Hansen, Sijin Wen, Shruti Jaiswal, Shitiz Sriwastava

**Affiliations:** 1 Government Medical College, Bhavnagar, Gujarat 364001, India; 2 B.J. Medical College, Ahmedabad 380016, India; 3 JSS Medical College, Mysore 570015, India; 4 Kathmandu Model College, Kathmandu 44600, Nepal; 5 College of Medicine, University of Illinois, Peoria, IL 61605, USA; 6 Tirunelveli Medical College, Tirunelveli 627011, India; 7 MGM Medical College, Indore 452001, India; 8 Department of Biostatistics, University of Michigan, Ann Arbor, MI 48109, USA; 9 Department of Biostatistics, West Virginia Clinical Transitional Sciences, Morgantown, WV 26506, USA; 10 Department of Neuro-Oncology, MD Anderson Cancer Center, Houston, TX 77030, USA; 11 Department of Neurology, Jackson T. Stephens Spine and Neurosciences Institute, University of Arkansas for Medical Sciences, Little Rock, AR 72205, USA

**Keywords:** BTKi, fenebrutinib, tolebrutinib, Bruton’s tyrosine kinase inhibitors, paramagnetic rim lesion

## Abstract

**Highlights:**

**What are the main findings?**

- BTK inhibitors significantly reduce relapse activity and MRI disease burden in multiple sclerosis.
- Hepatotoxicity remains a clinically relevant safety concern across trials.

**What are the implications of the main findings?**

- BTK inhibition offers a dual mechanism targeting peripheral immunity and CNS-compartmentalized inflammation.
- Long-term clinical adoption will depend on improved CNS penetration and safety optimization.

**Abstract:**

Background: Multiple sclerosis (MS), an autoimmune disease, involves peripheral immune activation followed by CNS inflammation in a compartmentalized manner. Although high-efficacy disease-modifying therapies (HE-DMTs) have been effective in suppressing relapses in MS patients, they fail to effectively target chronic microglial activation and smoldering lesions in MS patients. Bruton’s tyrosine kinase inhibitors (BTKis), which are orally active and capable of crossing the blood–brain barrier, have been found to be effective in modulating B cells and CNS-resident myeloid cells. Objective: The objective was to assess the efficacy and safety of Bruton’s tyrosine kinase inhibitors in patients with relapsing, secondary, and primary progressive MS. Methods: We performed a systematic review and meta-analysis according to the Cochrane and PRISMA guidelines (PROSPERO registration number: 1323474). We included randomized controlled trials (RCTs) that assessed fenebrutinib, evobrutinib, or tolebrutinib in adult MS patient populations. The main outcome measures were annualized relapse rate, MRI lesion activity, disability progression (EDSS), and hepatotoxicity. The quality of the included trials was assessed for bias by the RoB2 tool. Results: Six RCTs with 3616 participants were included. BTK inhibitors significantly reduced ARR compared with control therapy (pooled RR 0.24; 95% CI 0.15–0.39). MRI activity was reduced (mean difference −1.45 new/enlarging T2 lesions; 95% CI −2.08 to −0.82). Disability progression was unchanged in short-term relapsing MS trials. Serious hepatotoxicity was reported in 11.0% of BTKi-treated patients compared with 13.7% of control patients (pooled RR 0.80; 95% CI 0.66–0.96). However, increased transaminase elevations were reported in placebo-controlled trials, which indicates that hepatotoxicity remains a clinically relevant safety concern for the class. Conclusions: BTK inhibitors reduce inflammatory disease activity in relapsing MS and have emerging efficacy in progressive MS phenotypes; however, continued monitoring for hepatotoxicity is warranted. Optimization of CNS penetrance and pharmacologic selectivity may influence long-term clinical positioning.

## 1. Introduction

Multiple sclerosis (MS) is a chronic inflammatory demyelinating disorder of the central nervous system (CNS), characterized by immune-mediated damage to myelin, axons, and neurons. The disease arises when autoreactive immune cells infiltrate the CNS, leading to neurodegeneration and progressive neurological dysfunction. Although the precise pathophysiology of MS remains incompletely understood, it is widely accepted that the disease results from a complex interplay between genetic susceptibility and environmental triggers. These interactions contribute to a breakdown of immune tolerance to CNS antigens, ultimately triggering an aberrant inflammatory response [1].

While MS is initially driven by peripheral activation of the adaptive immune system, accumulating evidence indicates that disease progression is largely sustained by compartmentalized inflammation within the CNS. Following leukocyte migration across the blood–brain barrier (BBB), a self-perpetuating inflammatory environment is established. Within this compartment, activated microglia, CNS-resident macrophages, B cells, and T cells persist in the perivascular spaces, meninges, and parenchyma. In progressive forms of MS, ectopic lymphoid follicle-like structures composed of B and T lymphocytes are frequently observed in the meninges. These structures facilitate local antigen presentation, intrathecal antibody production, and sustained cytokine release, thereby maintaining chronic inflammation even in the context of reduced BBB permeability. Moreover, activated microglia play an important role in driving neurodegeneration by producing reactive oxygen species, nitric oxide, and pro-inflammatory cytokines, all of which contribute to chronic axonal injury. Collectively, these findings support the concept that MS is initiated by peripheral immune dysregulation but perpetuated by self-sustaining immune mechanisms within the CNS [2,3,4].

Current high-efficacy disease-modifying therapies (HE-DMTs) for multiple sclerosis (MS), including Ocrelizumab, Ofatumumab, Natalizumab, Alemtuzumab, Rituximab, and Cladribine, have improved clinical outcomes in patients with relapsing-remitting MS (RRMS). Early initiation of these therapies is associated with reductions in disease activity and relapse rates [5]. However, despite their efficacy in controlling acute inflammatory activity, these agents have limited impact on microglial activation, smoldering inflammation, and the progression of disability, particularly in progressive forms of the disease [6].

As discussed earlier, increasing evidence suggests that MS is sustained due to local immune mechanisms within the CNS. There is a formation of B-cell follicles within the meninges. These local immune mechanisms lead to slow-developing chronic active lesions. These limitations have prompted growing interest in therapeutic strategies that can target both adaptive and innate immune pathways, including those operating within the CNS compartment. In this context, recent advances in MS pharmacotherapy have focused on targeting Bruton’s tyrosine kinase (BTK), a key signaling molecule expressed in activated B cells and myeloid-lineage cells such as microglia. By modulating BTK activity, these emerging therapies hold promise for addressing both peripheral immune dysregulation and CNS-compartmentalized inflammation [5,6].

### 1.1. Biology of Bruton’s Tyrosine Kinase (BTK)

#### 1.1.1. BTK Structure and Signaling

Bruton’s tyrosine kinase (BTK) is a non-receptor cytoplasmic tyrosine kinase that plays a crucial role in intracellular signal transduction. Unlike receptor tyrosine kinases, non-receptor tyrosine kinases function within the cytoplasm, where they catalyze the transfer of phosphate groups from adenosine triphosphate (ATP) to specific tyrosine residues on target proteins, thereby modulating downstream signaling pathways. BTK belongs to the Tec family of kinases and serves as a central signaling mediator downstream of multiple immune receptors, including the B-cell receptor (BCR), Toll-like receptors (TLRs), Fc receptors (such as IgG-specific Fcγ receptors in macrophages and microglia and IgE-specific Fcε receptors in mast cells), and various cytokine receptors [7,8].

Although BTK was initially characterized for its role in B lymphocyte development and function, it is expressed across cells of the myeloid lineage (neutrophils, macrophages, dendritic cells, mast cells, and CNS-resident macrophages such as microglia). Increasing evidence indicates that BTK plays a role in regulating microglial activation and innate immune responses in the CNS, suggesting its involvement in the pathogenesis of neuroinflammatory disorders such as multiple sclerosis (MS) [9,10].

Structurally, BTK is a multidomain protein composed of five distinct regions, each contributing to its function and regulation:

- Pleckstrin homology (PH) domain: Located at the N-terminus, this domain binds phosphatidylinositol-3,4,5-trisphosphate (PIP3), facilitating the recruitment of BTK to the plasma membrane, where upstream signaling events occur.
- Tec homology (TH) domain: This region contributes to structural stability and supports interactions with adaptor proteins involved in signal transduction.
- Src homology 3 (SH3) domain: Mediates protein–protein interactions by binding to proline-rich motifs in partner proteins.
- Src homology 2 (SH2) domain: This domain recognizes and binds phosphorylated tyrosine residues on signaling proteins, enabling the assembly of signaling complexes and contributing to the regulation of BTK activation.
- Kinase (SH1) domain: Located at the C-terminus, this catalytic domain is responsible for BTK’s enzymatic activity, phosphorylating target proteins on tyrosine residues and thereby propagating intracellular signaling pathways that regulate immune cell activation, proliferation, and survival [9].

#### 1.1.2. BTK-Dependent Pathways

BTK is activated downstream of multiple immune receptors and undergoes conformational changes during this process. In its resting state, BTK is maintained in an autoinhibited configuration through intramolecular interactions involving the SH2 domain, while the PH domain remains unbound to membrane phospholipids. Upon receptor stimulation, BTK activation proceeds through a sequence of events:

1. Engagement of immune receptors such as the B-cell receptor (BCR), Toll-like receptors (TLRs), Fc receptors, or cytokine and chemokine receptors by their respective ligands initiates intracellular signaling cascades.
2. This stimulation activates phosphoinositide 3-kinase (PI3K), which catalyzes the conversion of phosphatidylinositol-4,5-bisphosphate (PIP2) into phosphatidylinositol-3,4,5-trisphosphate (PIP3).
3. The PH domain of BTK exhibits high affinity for PIP3, promoting its recruitment to the plasma membrane and inducing conformational changes that expose the kinase domain.
4. BTK is subsequently phosphorylated at tyrosine residue Y551 by Src-family kinases (e.g., Lyn, Fyn, Hck, or Syk-associated kinases), resulting in a substantial increase in its catalytic activity.
5. Full activation is achieved through autophosphorylation at tyrosine residue Y223, stabilizing BTK in its active conformation [9,11].

Once activated, BTK propagates intracellular signaling primarily through phosphorylation of phospholipase C gamma 2 (PLCγ2). In microglial cells, however, BTK signaling extends beyond PLCγ2 activation to include the induction of key transcription factors such as nuclear factor kappa B (NF-κB) [12]. Additionally, BTK has been implicated in the phosphorylation of Toll/interleukin-1 receptor domain-containing adaptor protein (TIRAP), thereby facilitating activation of mitogen-activated protein kinase (MAPK) pathways and further enhancing NF-κB-mediated transcriptional responses [13].

These downstream signaling events collectively regulate immune cell function by modulating gene expression, cellular metabolism, and survival pathways. BTK influences the following processes:

- Cell proliferation through activation of MAPK signaling pathways.
- Protein synthesis and metabolic regulation via the mTOR pathway.
- Calcium signaling and NFAT activation mediated by PLCγ2.
- Cell survival and inflammatory responses driven by NF-κB signaling.

Within the CNS, BTK plays an important role in microglial activation. Activation of BTK in microglia enhances NF-κB signaling, leading to the release of pro-inflammatory cytokines, activation of inflammasomes, and amplification of neuroinflammatory processes [9]. Thus, in microglial cells, BTK functions as a link between innate immune receptor activation and the propagation of chronic inflammation. In contrast to CD20-targeting therapies, which primarily modulate peripheral B-cell activity, BTK inhibitors can target both peripheral immune responses and compartmentalized CNS inflammation. This dual mechanism underlies their emerging therapeutic relevance in neuroinflammatory conditions such as multiple sclerosis [12].

### 1.2. Role of BTK-Expressing Cells in MS Pathogenesis

#### 1.2.1. B Cells Beyond Antibody Production

B cells contribute to the pathogenesis of autoimmune diseases through both antibody-dependent and antibody-independent mechanisms. In MS, evidence suggests that the role of B cells in relapsing disease activity is largely driven by their antibody-independent functions, particularly within the peripheral immune system. Activated B cells can act as potent antigen-presenting cells, promoting aberrant activation of pro-inflammatory T cells and thereby amplifying immune-mediated damage. Clinically patients with MS relapses show increased frequency of circulating memory B cells. These cells have an increased CD40 and HLA-DR expression, reflecting an enhanced capacity for antigen presentation to CD4^+^ T lymphocytes. This suggests antigen-presenting function contributes to the propagation of autoreactive T-cell responses and sustained inflammation [3,10,14].

In addition to their role in antigen presentation, memory B cells contribute to MS pathogenesis through the production of pro-inflammatory cytokines. Compared with B cells from healthy individuals, memory B-cell subsets in MS patients secrete higher levels of cytokines including TNF-α, IL-6, and GM-CSF. These cytokines play critical roles in modulating immune responses: IL-6 inhibits the differentiation of regulatory T cells while promoting B-cell survival through autocrine signaling; TNF-α enhances B-cell activation and supports germinal center formation; and GM-CSF stimulates the activation of macrophages and microglia, thereby contributing to localized CNS inflammation. Collectively, the multifaceted role of memory B cells drives both peripheral immune dysregulation and central neuroinflammatory processes in MS [14].

The development of structures analogous to secondary lymphoid organs has also been reported in meninges at sites of chronic inflammation, serving as a reservoir for auto-reactive B and T cell reactivation. Ectopic lymphoid tissues (ELTs) have been demonstrated in the meninges of approximately 40% of secondary progressive multiple sclerosis (SPMS) patients. Formation of ELTs requires persistent antigen exposure, chronic inflammation, and sustained B- and T-cell infiltration, along with continuous cytokine signaling. The most critical cytokine for ELT formation is lymphotoxin alpha. With sustained exposure to LTα and TNF, meningeal fibroblast-like cells begin to acquire functional characteristics of lymphoid tissue organizer cells (LTO). These cells express CXCL13 and CCL19 and produce IL-6, which recruits B and T cells. These recruited cells are also organized in places by chemokines. Once these ELTs are formed, they sustain themselves via IL-6 and TNF-alpha [4,6].

Chronic inflammation in MS is associated with the formation of structures resembling secondary lymphoid organs within the meninges. These ectopic lymphoid tissues (ELTs) act as niches that support the reactivation and persistence of autoreactive B and T lymphocytes. ELTs have been identified in around 40% of patients with secondary progressive multiple sclerosis (SPMS). ELTs are driven by sustained antigenic stimulation, chronic inflammatory signaling, and continuous infiltration of B and T cells. Among the cytokines involved, LTα plays a key role in initiating their formation. Prolonged exposure to LTα and TNF-α induces meningeal stromal cells, particularly fibroblast-like cells, to acquire features of lymphoid tissue organizer (LTo) cells. These transformed cells produce chemokines (CXCL13 and CCL19) as well as IL-6, which promote the recruitment, retention, and spatial organization of lymphocytes within the meninges. Once established, ELTs become self-sustaining through ongoing cytokine production (IL-6 and TNF-α), maintaining a localized inflammatory microenvironment, which contributes to persistent CNS inflammation and progressive neurodegeneration in MS [4,6].

#### 1.2.2. Myeloid Cells and Microglia

In chronic active MS lesions, inflammation and axonal injury are localized at the lesion margins, in contrast to early lesions where inflammation is more diffuse. These lesion rims are composed of HLA-DR^+^-activated microglia and macrophages, with few parenchymal T cells and minimal B-cell presence. Notably, the distribution of myeloid cell infiltration closely corresponds to areas of axonal transection, highlighting their central role in ongoing tissue damage.

A defining feature of these chronic active lesions is the accumulation of iron within rim-associated myeloid cells. This iron deposition gives rise to “iron rims,” which can be visualized using iron-sensitive magnetic resonance imaging as paramagnetic rim lesions (PRLs). PRLs are recognized as imaging biomarkers of persistent, smoldering inflammation [6,7].

Microglia within chronic lesions remain chronically activated due to continuous exposure to myelin debris, pro-inflammatory cytokines, oxidative stress, and inflammatory signals originating from ELTs. Activated microglia contribute to neurodegeneration through both direct and indirect mechanisms. Direct axonal injury is mediated by the release of ROS, which damage cellular membranes and DNA; glutamate, which induces excitotoxicity; and pro-inflammatory cytokines, which disrupt axonal transport. In parallel, microglia promote mitochondrial dysfunction through nitric oxide production and iron-mediated oxidative stress, leading to impaired ATP generation and increased metabolic demand within axons.

These processes culminate in the formation of chronic active, or “smoldering,” lesions, characterized by an inactive demyelinated core surrounded by a rim of iron-laden, activated microglia. Over time, expansion of this rim drives ongoing neurodegeneration and contributes to progressive clinical decline in MS [2,6].

BTK signaling is crucial in regulating microglial function within these lesions. BTK is involved in phagocytosis, cytoskeletal remodeling, actin polymerization, and phagosome formation. While phagocytosis may serve protective roles, excessive or dysregulated activity can amplify inflammatory responses. BTK activation also promotes the release of pro-inflammatory cytokines (TNF, IL-1, IL-6, and GM-CSF), which contribute to axonal injury and further stimulation [9,12].

## 2. Methods

**Figure 1:**
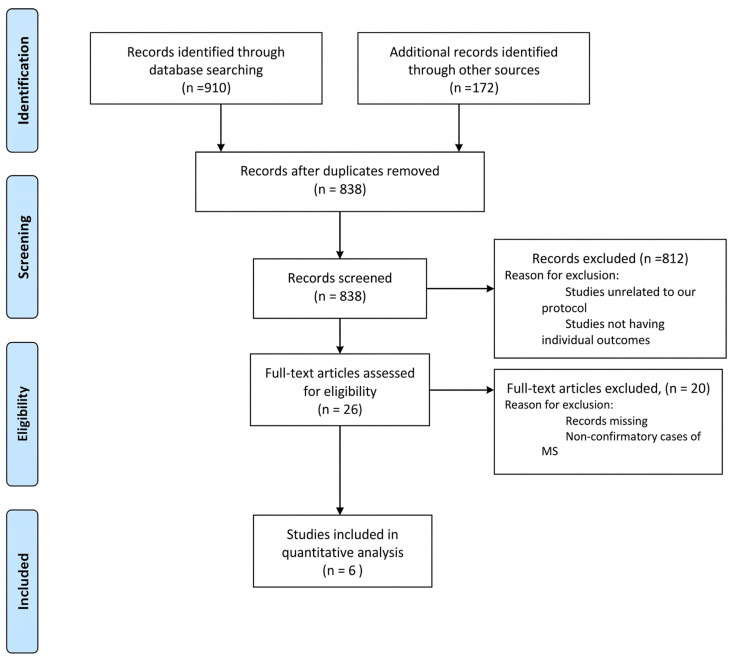
Preferred Reporting Items for Systematic Reviews and Meta-Analyses (Prisma) Flow Diagram.

This systematic review and meta-analysis was conducted in accordance with the PRISMA guidelines and the Cochrane Handbook for Systematic Reviews (Figure 1) [Supplementary Materials Table S1]. The study protocol was prospectively registered in PROSPERO (Registration ID: 1323474). A comprehensive literature search was performed across four electronic databases: PubMed (MEDLINE), Web of Science, Scopus, and Google Scholar. Studies published between January 2020 and January 2026 were considered. The search strategy combined Medical Subject Headings (MeSH) terms and keywords related to multiple sclerosis and Bruton’s tyrosine kinase (BTK) inhibitors, including: “multiple sclerosis,” “relapsing-remitting multiple sclerosis,” “secondary progressive multiple sclerosis,” “primary progressive multiple sclerosis,” “BTK inhibitor,” “Bruton’s tyrosine kinase inhibitor,” “Fenebrutinib,” “Evobrutinib,” and “Tolebrutinib.” Reference lists of relevant studies and reviews were also manually screened to ensure comprehensive study identification.

### 2.1. Eligibility Criteria

Studies were included if they met the following criteria: (1) Randomized controlled trials (RCTs) evaluating the efficacy and/or safety of BTK inhibitors in multiple sclerosis; (2) Studies examining adult participants diagnosed with relapsing-remitting MS (RRMS), secondary progressive MS (SPMS; active or non-active), or primary progressive MS (PPMS); (3) Studies reporting at least one relevant clinical or radiological outcome related to disease activity or progression. We excluded articles that were not published in the English language; were not in peer-reviewed journals, including conference abstracts, book chapters, or non-indexed reports; used secondary research designs, including reviews, systematic reviews, meta-analyses, protocols, editorials, commentaries, hypotheses, and opinion papers; and were non-randomized clinical trials in animal models.

### 2.2. Selection Process

Records identified from the database search were imported into EndNote for duplicate removal. Three authors screened the available literature by title and abstract. Studies from the title and abstract selection were retrieved and screened by full text following our inclusion criteria. Conflict and disagreement between authors were resolved through discussion and consultation with the senior author. We additionally performed a manual search of the reference list of relevant reviews and included studies to ensure comprehensive study selection.

### 2.3. Data Extraction

Data were independently extracted by three reviewers using a pre-piloted standardized Excel form, with a fourth reviewer verifying accuracy and resolving discrepancies. The extracted domains included: (1) Study characteristics: study design, population, intervention and dosage, comparator, follow-up duration, and key findings; (2) Baseline characteristics: sample size, age, sex distribution, MS subtype, disease duration, baseline Expanded Disability Status Scale (EDSS) score, and number of gadolinium-enhancing lesions; (3) Outcomes: changes in EDSS scores (e.g., at 3 and 6 months), number of new T1 gadolinium-enhancing and T2 lesions on MRI, percentage brain volume change, and safety outcomes showing hepatotoxicity.

### 2.4. Quality Assessment

The risk of bias for included randomized controlled trials was assessed using the Cochrane Risk of Bias 2 (RoB 2) tool. This tool evaluates five domains: randomization process, deviations from intended interventions, missing outcome data, outcome measurement, and selective reporting. Studies were categorized as “low risk,” “some concerns,” or “high risk” based on domain-level assessments. Where applicable, crossover trials were assessed using the appropriate RoB 2 extension. Visualization of bias assessments was performed using the Robvis tool.

### 2.5. Statistical Analyses

Statistical analyses were conducted using R software (version 3.6.3). Descriptive statistics are used to summarize study characteristics and outcomes. Categorical variables are presented as frequencies and percentages, while continuous variables are summarized as means or medians with corresponding ranges. Where appropriate, pooled analyses were performed across treatment groups. For categorical outcomes, contingency tables were constructed and analyzed using the chi-square test.

## 3. Results

### 3.1. Study Selection

A comprehensive database search identified a total of 1082 records. After removal of 244 duplicates, 838 studies remained for screening. Of these, 812 articles were excluded due to irrelevance, including observational studies, review articles, preclinical or non-human studies, and other non-eligible publication types (e.g., letters and editorials). Full-text assessment was conducted for 25 studies, of which 19 were excluded due to insufficient or non-relevant data for the predefined outcomes. Ultimately, six randomized controlled trials (RCTs) [15,16,17,18,19,20] met the inclusion criteria and were included in the systematic review and meta-analysis (Table 1).

### 3.2. Study Characteristics

A total of six randomized controlled trials evaluating Bruton’s tyrosine kinase (BTK) inhibitors (fenebrutinib, tolebrutinib, and evobrutinib) were included in this analysis. The studies enrolled adult participants aged 18–65 years, with sample sizes ranging from 33 to 1131 individuals. The majority of participants were diagnosed with RRMS, although some trials also included patients with SPMS. All investigated BTK inhibitors were administered orally. Comparator groups varied across studies: four trials used placebo controls [15,16,19,20], while two trials employed an active comparator, teriflunomide [17,18]. Dosing regimens differed considerably depending on the specific agent and study design, ranging from 5 mg to 200 mg daily.

### 3.3. Safety: Serious Hepatotoxicity

**Figure 2:**
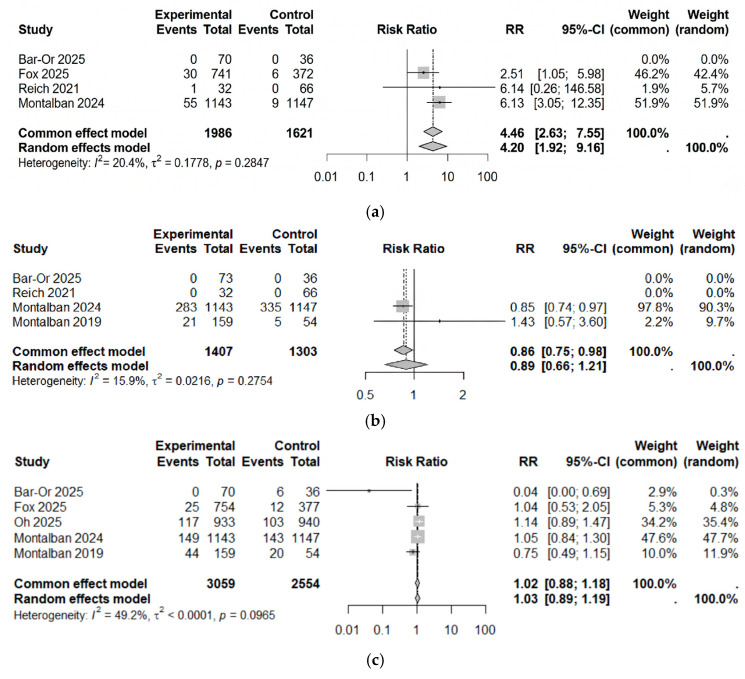
(**a**) Forest Plot of Serious Hepatotoxicity in Patients Receiving BTK Inhibitors versus Control [15,18,19,20]. (**b**) Forest Plot of Mild-to-Moderate Hepatotoxicity in Patients Receiving BTK Inhibitors versus Control [15,16,18,20]. (**c**) Forest Plot of Relapse Rates in Patients Receiving BTK Inhibitors versus Control [16,17,18,19,20].

In placebo-controlled trials, serious hepatotoxicity (defined as liver-related adverse events with elevated liver enzymes and/or bilirubin) occurred in 51 of 900 participants (5.7%) in the BTK inhibitor group and in 11 of 426 participants (2.6%) in the placebo group (RR, 2.19; 95% CI, 1.13–4.25) (Figure 2a). In trials with teriflunomide as an active comparator, hepatotoxicity was reported in 173 of 1143 participants (15.1%) receiving BTK inhibitors and in 204 of 1147 participants (17.8%) receiving teriflunomide (RR, 0.85; 95% CI, 0.71–1.01). Mild-to-moderate hepatotoxicity was reported with an RR of 0.86 (95% CI, 0.75–0.98) in participants receiving BTK inhibitors compared with control (Figure 2b).

### 3.4. Efficacy: Relapse Outcomes

Relapse outcomes were reported in three trials. Annualized relapse rates were 0.168 in the BTK inhibitor group and 0.634 in the control group in Bar-Or (2025), 0.23 versus 2.12 in Reich (2021), and 0.13 versus 0.37 in Montalban (2019; 75 mg once daily) [15,16,20]. The pooled relative risk for relapse was 0.24 (95% CI, 0.15–0.39). Figure 2c shows a forest plot of relapse rates in patients receiving BTKi.

### 3.5. Magnetic Resonance Imaging (MRI) Outcomes

MRI outcomes were reported in the included studies. Mean numbers of new or enlarging T2 lesions were 0.077 in the BTK inhibitor group and 0.245 in the control group in Bar-Or (2025), 0.13 versus 1.4 in Reich (2021), and 1.69 versus 3.85 in Montalban (2019). The pooled mean difference was −1.45 lesions (95% CI, −2.08 to −0.82) [15,16,20].

### 3.6. Disability Outcomes

Baseline Expanded Disability Status Scale (EDSS) scores ranged from 2.3 to 8.3 across the included trials. Reporting of post-treatment EDSS scores was inconsistent across the included trials, with most studies not providing comparative data (Table 1). Among the included BTK inhibitor trials, the Montalban 2019 study reported no significant difference in EDSS change from baseline, while the FENopta extension study reported stable EDSS scores over follow-up; however, numerical post-treatment EDSS values were not provided.

## 4. Discussion

BTKis have been investigated as a potential therapeutic approach in MS to address the progression of disability associated with compartmentalized, chronic neuroinflammation [21,22]. Unlike anti-CD20 monoclonal antibodies, which deplete circulating B cells and have limited penetration across the blood–brain barrier (BBB), BTKis are small-molecule agents with the capacity to access the central nervous system (CNS) [21].

BTKis are proposed to effect through both peripheral and central mechanisms. In the peripheral immune system, they modulate B-cell function, including activation, proliferation, and antigen presentation, without complete depletion, thereby maintaining baseline immune function and immunoglobulin levels [23]. Within the CNS, BTKis act on microglia and macrophages by inhibiting signaling pathways mediated by Fc receptors and Toll-like receptors, which are involved in inflammatory activation. This mechanism has been associated with modulation of chronic active lesions, including slowly expanding lesions (SELs), which contribute to progression independent of relapse activity (PIRA) [21,24]. Preclinical studies showed that BTK inhibition is associated with reduced microglial activation, enhanced clearance of myelin debris, and effects on remyelination processes [25]. Phase 2 clinical trials have reported reductions in gadolinium-enhancing (Gd+) T1 lesions and changes in SEL-related imaging outcomes at selected doses [15,16].

Phase 3 trials evaluating evobrutinib (EVOLUTION) and tolebrutinib (GEMINI) in relapsing multiple MS did not demonstrate superiority over the active comparator, teriflunomide, in reducing annualized relapse rates (ARRs), despite positive findings in earlier phase studies [17,18]. Several factors have been proposed to account for these findings. Unlike earlier trials, Phase 3 studies employed an active comparator rather than placebo. In these trials, teriflunomide was associated with low on-treatment relapse rates (approximately 0.11–0.14), which reduced the ability to detect differences between treatment groups [17,18]. In addition, pharmacokinetic data indicated that evobrutinib achieved CSF concentrations of approximately 3.2 ng/mL (7.45 nM), which were below the estimated concentration required for high-level target engagement in the CNS. This exposure was lower than the reported half-maximal inhibitory concentration (IC50) of 33.5 nM, suggesting limited inhibition of compartmentalized CNS inflammation at the administered dose [18,26]. The mechanism of action of BTK inhibitors, which involves modulation rather than depletion of B cells, has also been considered in relation to their effects on relapse activity. BTK inhibition may have differential effects on peripheral immune responses compared with established therapies that directly reduce circulating B-cell populations [17,18]. Safety findings were also reported in these trials. Elevations in alanine aminotransferase (ALT) ≥ 5× the upper limit of normal (ULN) occurred in 5.0% of participants receiving evobrutinib, while tolebrutinib was associated with ALT elevations exceeding 20× ULN in 0.5% of participants [18,19]. Reports of drug-induced liver injury (DILI) led to regulatory actions, including temporary clinical holds by the U.S. Food and Drug Administration (FDA) [27].

Subsequent clinical data have provided information regarding the therapeutic role of BTKis in MS. Evidence from later-phase trials has evaluated their use across different MS subtypes, with attention to pharmacokinetic properties and patient populations. In progressive MS, BTK inhibitors have been studied in populations characterized by compartmentalized CNS inflammation. In the Phase 3 HERCULES trial, tolebrutinib, which demonstrates higher CNS penetration relative to earlier compounds, was associated with a 31% reduction in 6-month confirmed disability progression compared with placebo in patients with non-relapsing secondary progressive MS. Based on these findings, tolebrutinib received Breakthrough Therapy designation from the U.S. FDA [19]. In addition, results from the Phase 3 FENtrepid trial indicated that fenebrutinib met its primary endpoint in patients with primary progressive MS (PPMS), demonstrating noninferiority to ocrelizumab with respect to 12-month composite confirmed disability progression (cCDP12), with a reported risk reduction of 12% [28].

In relapsing MS, results from the Phase 3 FENhance 2 trial demonstrated that fenebrutinib reduced relapse rates compared with teriflunomide. Earlier Phase 2 data from the FENopta trials also reported reductions in relapse rates compared with placebo [18,20]. Fenebrutinib is a noncovalent, reversible BTK inhibitor with higher selectivity for BTK relative to other kinases, which distinguishes it from earlier compounds evaluated in this class.

Safety monitoring approaches have also been modified in response to earlier observations of hepatotoxicity. Clinical trial protocols have incorporated enhanced liver function monitoring to detect elevations in transaminases during treatment [18,19]. While hepatotoxicity-related mortality and treatment discontinuation were rare across the included studies, severe hepatic events were clinically significant when they occurred. For instance, in the evolutionRMS trials, three participants met Hy’s law criteria and recovered following treatment discontinuation. However, 2 deaths did occur during the trial, which were not considered treatment-related [18]. In the phase 3 tolebrutinib study, one participant underwent liver transplantation and subsequently died from postoperative complications following tolebrutinib-related severe liver injury [19]. This highlights the importance of liver-function monitoring during BTK inhibitor therapy.

The pooled relative risk of 0.24 for relapse observed in this meta-analysis highlights the potent clinical efficacy of BTK inhibitors, establishing this class as a high-efficacy disease-modifying therapy. While individual Phase 3 trials have presented challenges in demonstrating superiority against active comparators, these aggregate data show that the class possesses strong intrinsic therapeutic activity. This clinical signal suggests that the initial trial results may have been constrained by trial design rather than a deficiency in efficacy, providing a robust rationale for the continued clinical advancement of BTK inhibitors. Further studies might cement the position of BTK inhibitors as an effective class by establishing success in progressive MS subtypes and refining the pharmacological features required to treat relapsing MS.

### 4.1. Study Limitations

This analysis has several limitations. The number of randomized controlled trials evaluating BTKi in MS is limited, and the included studies exhibited heterogeneity in dosing regimens, comparator therapies, and patient populations. The majority of participants were diagnosed with relapsing MS (RMS), which limits the applicability of our findings to progressive disease subtypes. Because most studies did not publish post-treatment EDSS scores or mean changes in EDSS from baseline, it is difficult to interpret disability results across BTK inhibitor trials. The lack of EDSS data limits direct quantitative comparison of disability trajectories among studies. Instead, few studies evaluated disability through confirmed disability worsening (CDW) or confirmed disability progression (CDP) endpoints. The reporting of CDW and CDP outcomes was largely confined to trials with longer follow-up durations, whereas shorter phase 2 studies primarily focused on MRI and relapse-related endpoints. Given the slow accumulation of disability in multiple sclerosis, shorter follow-up periods may be insufficient to detect meaningful changes in disability status. Consequently, disability outcomes were less reported in these studies. In addition, differences in safety monitoring protocols across trials may have affected the detection and reporting of hepatotoxicity events.

### 4.2. Clinical Implications

The findings indicate that BTK inhibitors are associated with reductions in relapse rates and MRI measures of disease activity in MS. Effects on disability progression were limited within the duration of the included trials. BTK inhibitors have been evaluated in both relapsing and progressive forms of MS, including populations with compartmentalized CNS inflammation. Liver function monitoring remains a component of clinical evaluation due to reported hepatotoxicity events.

### 4.3. Future Research

Further research is required to evaluate the long-term effects of BTK inhibitors on disability progression, particularly in progressive MS subtypes. Additional studies may examine the impact of pharmacological properties, including selectivity and CNS penetration, on clinical outcomes. Comparative trials and real-world studies may provide additional information on long-term safety and effectiveness in broader patient populations.

## Figures and Tables

**Table 1:** Summary of Included Randomized Controlled Trials of BTK Inhibitors in Multiple Sclerosis.

Study 2025	Drug	Design	MS Subtype	N (BTKi)	Comparator	Dose/Route	Primary Efficacy (BTKi vs. Comp)	ARR (BTKi vs. Comp)	MRI Outcomes	EDSS (Baseline > Follow-Up)	Safety Findings	F/u Time (Wk)
Bar-Or 2025 [20]	Fenebrutinib	RCT, double-blind	RRMS/RMS (106); SPMS (3)	70	Placebo	200 mg BID (oral)	0.168 vs. 0.634	0 vs. 0.18	0.077 vs. 0.245 (69% reduction)	2.5 → EDSS used only as inclusion	0 vs. 0 (4/73 total AEs noted)	48
Fox 2025 [19]	Tolebrutinib	RCT, double-blind	SPMS (1131)	754	Placebo	60 mg OD (oral)	1.84 vs. 2.95	0.033 vs. 0.032	Brain volume loss: 0.37%; 21% reduction in atrophy	5.5 → NR	30/741 vs. 6/372	Median: 133
Reich 2021 [15]	Tolebrutinib	Phase 2 RCT, double-blind	RRMS/RMS (128); SPMS (2)	33 (5 mg), 32 (15 mg), 33 (30 mg), 32 (60 mg)	Placebo	5, 15, 30, 60 mg (oral)	0.23 vs. 2.12	NR	0.13 vs. 1.4	2.3 → NR	1/32 vs. 0	16
Oh 2025 (GEMINI 1 & 2) [17]	Tolebrutinib	Two Phase 3 RCTs, double-blind	RMS only (G1: 974; G2: 899)	G1: 486; G2: 447	Teriflunomide	60 mg OD (oral)	G1: 5.611 vs. 5.175; G2: 5.092 vs. 4.369	G1: 0.13 vs. 0.11; G2: 0.12 vs. 0.11	G1: 0.53 vs. 0.285; G2: 0.46 vs. 0.217	≤5.5 → Median: G1 15.38 vs. 17.97; G2 15.12 vs. 12.14	Not clearly reported	Median: 139
Montalban 2024 (EvolutionRMS1/2) [18]	Evobrutinib	Two Phase 3 RCTs, double-blind	RMS only	RMS1: 560; RMS2: 583	Teriflunomide	45 mg BID (oral)	NR	RMS1: 0.15 vs. 0.14; RMS2: 0.11 vs. 0.11	NR	≤5.5 → NR	55 vs. 9 (ALT 173; AST 110 elevations)	156
Montalban 2019 [16]	Evobrutinib	Phase 2 RCT, double-blind	RRMS/RMS (228); SPMS (33)	159	Placebo	25 mg OD; 75 mg OD; 75 mg BID (oral)	4.06 (25 mg), 1.69 (75 mg OD), 1.15 (75 mg BID) vs. 3.85	Wk24: 0.57, 0.13, 0.08 vs. placebo; Wk48: 0.52, 0.25, 0.11	No significant reduction (RR = 1.45 for 25 mg); ~70% reduction higher dose	Mean 3.3 → NR	ALT: 3, 6, 5; AST: 1, 2, 4	48

**BTKi:** Bruton’s Tyrosine Kinase inhibitor; **MS:** Multiple Sclerosis; **RCT:** Randomized Controlled Trial; **RRMS:** Relapsing-Remitting Multiple Sclerosis; **RMS:** Relapsing Multiple Sclerosis; **SPMS:** Secondary Progressive Multiple Sclerosis; **N:** Number of participants; **Comp:** Comparator; **ARR:** Annualized Relapse Rate; **MRI:** Magnetic Resonance Imaging; **EDSS:** Expanded Disability Status Scale; **BID:** Twice a day; **OD:** Once a day; **NR:** Not Reported; **G1, G2:** GEMINI 1, GEMINI 2; **ALT:** Alanine Aminotransferase; **AST:** Aspartate Aminotransferase; **Wk:** Week; **RR:** Relative Risk; **F/u:** Follow-up; **OLE:** Open-Label Extension.

## Data Availability

No new data were created or analyzed in this study.

## Supplementary Materials

The following supporting information can be downloaded at: https://www.mdpi.com/article/10.3390/brainsci16060634/s1, Table S1: PRISMA 2009 Checklist [29].

## Abbreviations

ALT	Alanine Aminotransferase
ARR	Annualized Relapse Rate
ATP	Adenosine Triphosphate
BBB	Blood–Brain Barrier
BCR	B-cell Receptor
BTK	Tyrosine Kinase
BTKi	Bruton Tyrosine Kinase Inhibitors
CCL9	C-C Motif Chemokine Ligand 9
CD20	Cluster of Differentiation 20
CDW	Confirmed Disability Worsening
CDP	Confirmed Disability Progression
CI	Confidence Interval
CNS	Central Nervous System
CSF	Cerebrospinal Fluid
cCDP12	Composite Confirmed Disability Progression (at 12 months)
CXCL13	C-X-C Motif Chemokine Ligand 13
DILI	Drug-Induced Liver Injuries
EDSS	Expanded Disability Status Scale
ELTs	Ectopic Lymphoid Tissues
FDA	Food and Drug Administration
Fcε	Fragment crystallizable epsilon
Fcγ	Fragment crystallizable gamma
Gd+	Gadolinium-enhancing (lesions)
GM-CSF	Granulocyte-Macrophage Colony-Stimulating Factor
HEDMTs	High-Efficacy Disease-Modifying Therapies
HLA-DR	Human Leukocyte Antigen-DR isotype
IC50	Half-Maximal Inhibitory Concentration
IC90	Inhibitory Concentration at 90%
IgE	Immunoglobulin E
IgG	Immunoglobulin G
IL-1	Interleukin-1
IL-6	Interleukin-6
LTO	Lymphoid Tissue Organizer cells
LTα	Lymphotoxin alpha
MAPs	Mitogen-Activated Protein Kinases
MRI	Magnetic Resonance Imaging
mTOR	Mechanistic Target of Rapamycin
NF-	Nuclear factor kappa-light-chain-enhancer of activated B cells
κB	Nuclear Factor of Activated T-cells
NFAT	Non-Relapsing Secondary Progressive Multiple Sclerosis
nrSPMS	Pleckstrin Homology (domain)
PH	Phosphatidylinositol 4,5-bisphosphate
PIP2	Phosphatidyl Inositol Triphosphate Progression
PIP3	Independent of Relapse Activity Phospholipase
PIRA	C gamma 2
PLCγ2	Primary Progressive Multiple Sclerosis
PPMS	Preferred Reporting Items for Systematic Reviews and
PRISA	Meta-Analyses
PRLs	Paramagnetic Rim Lesions
PROSPER	International Prospective Register of Systematic Reviews
RCTs	Randomized Controlled Trials
RMS	Relapsing Multiple Sclerosis
RoB2	Risk of Bias 2
ROS	Reactive Oxygen Species
RR	Relative Risk
RRMS	Relapsing-Remitting Multiple Sclerosis
SELs	Slowly Expanding Lesions
SH1	Kinase (SH1) domain
SH2	SH2 domain
SH3	SH3 domain
SPMS	Secondary Progressive Multiple Sclerosis
TIRP	Toll-interleukin 1 receptor (TIR) domain-containing adaptor protein
TH	Tec Homology
TNF	Tumor Necrosis Factor
TNF-α	Tumor Necrosis Factor-alpha
ULN	Upper Limit of Normal
