# Supplementary material for: Emerging Role of BTK Inhibitors in Multiple Sclerosis: From Immunobiology to Clinical Translation
Source: Brain Sci. 2026 Jun 12;16(6):634. doi: 10.3390/brainsci16060634 (PMC13297389; doi:10.3390/brainsci16060634)
Supplement: Supplementary file 1 [file brainsci-16-00634-s001.zip › brainsci-4355962-supplementary.pdf]

Section and Topic	Item #	Checklist item	Location where item is reported
**TITLE**			
Title	1	Identify the report as a systematic review.	Page 1, Identified as a systematic review and meta-analysis in the title
**ABSTRACT**			
Abstract	2	See the PRISMA 2020 for Abstracts checklist.	Page 2 Structured abstract following PRISMA guidelines (Objectives, Methods, Results, Conclusion) reported in Abstract section
**INTRODUCTION**			
Rationale	3	Describe the rationale for the review in the context of existing knowledge.	Page 5-8 Introduction section (Section 1.0)
Objectives	4	Provide an explicit statement of the objective(s) or question(s) the review addresses.	Page 9 Clearly stated in Abstract and Methods section
**METHODS**			
Eligibility criteria	5	Specify the inclusion and exclusion criteria for the review and how studies were grouped for the syntheses.	Page 12 Section 2.1 Eligibility Criteria
Information sources	6	Specify all databases, registers, websites, organisations, reference lists and other sources searched or consulted to identify studies. Specify the date when each source was last searched or consulted.	Page 12 Section 2.0 Methods (Databases: PubMed, Web of Science, Scopus, Google Scholar; last searched January 2026)
Search strategy	7	Present the full search strategies for all databases, registers and websites, including any filters and limits used.	Page 11-12 Search strategy described in Section 2.0 Methods
Selection process	8	Specify the methods used to decide whether a study met the inclusion criteria of the review, including how many reviewers screened each record and each report retrieved, whether they worked independently, and if applicable, details of automation tools used in the process.	Section 2.2 Selection Process (three reviewers independently screened studies)

Section and Topic	Item #	Checklist item	Location where item is reported
Data collection process	9	Specify the methods used to collect data from reports, including how many reviewers collected data from each report, whether they worked independently, any processes for obtaining or confirming data from study investigators, and if applicable, details of automation tools used in the process.	Section 2.3 Data Extraction (three reviewers with fourth reviewer verification)
Data items	10a	List and define all outcomes for which data were sought. Specify whether all results that were compatible with each outcome domain in each study were sought (e.g. for all measures, time points, analyses), and if not, the methods used to decide which results to collect.	Section 2.3 Data Extraction (EDSS, MRI lesions, ARR, hepatotoxicity)
	10b	List and define all other variables for which data were sought (e.g. participant and intervention characteristics, funding sources). Describe any assumptions made about any missing or unclear information.	Section 2.3 Data Extraction (study characteristics, baseline demographics, interventions)
Study risk of bias assessment	11	Specify the methods used to assess risk of bias in the included studies, including details of the tool(s) used, how many reviewers assessed each study and whether they worked independently, and if applicable, details of automation tools used in the process.	Section 2.4 Quality Assessment (RoB 2 tool; visualization via Robvis)
Effect measures	12	Specify for each outcome the effect measure(s) (e.g. risk ratio, mean difference) used in the synthesis or presentation of results.	Effect measures included relative risk (RR) for dichotomous outcomes and mean differences for continuous outcomes, with 95% confidence intervals (Section 2.5 Statistical Analyses).
Synthesis methods	13a	Describe the processes used to decide which studies were eligible for each synthesis (e.g. tabulating the study intervention characteristics and comparing against the planned groups for each synthesis (item #5)).	Section 2.5 Statistical Analysis (grouping based on intervention and outcomes)
	13b	Describe any methods required to prepare the data for presentation or synthesis, such as handling of missing summary statistics, or data conversions.	Methods for data preparation are not explicitly reported.
	13c	Describe any methods used to tabulate or visually display results of individual studies and syntheses.	Results presented using tables and forest plots (Figures 2.1–2.3)
	13d	Describe any methods used to synthesize results and provide a rationale for the choice(s). If meta-analysis was performed, describe the model(s), method(s) to identify the presence and extent of statistical heterogeneity, and software package(s) used.	Section 2.5 Statistical Analysis (R software; pooled analysis

Section and Topic	Item #	Checklist item	Location where item is reported
			performed; chi-square test used)
	13e	Describe any methods used to explore possible causes of heterogeneity among study results (e.g. subgroup analysis, meta-regression).	Not performed
	13f	Describe any sensitivity analyses conducted to assess robustness of the synthesized results.	Sensitivity analysis not performed
Reporting bias assessment	14	Describe any methods used to assess risk of bias due to missing results in a synthesis (arising from reporting biases).	Reporting bias was not formally assessed due to the limited number of included studies.
Certainty assessment	15	Describe any methods used to assess certainty (or confidence) in the body of evidence for an outcome.	Certainty of evidence was not assessed using GRADE.
**RESULTS**			
Study selection	16a	Describe the results of the search and selection process, from the number of records identified in the search to the number of studies included in the review, ideally using a flow diagram.	Page 13 Section 3.1 Study Selection and PRISMA flow diagram
	16b	Cite studies that might appear to meet the inclusion criteria, but which were excluded, and explain why they were excluded.	Page 13-14 Reasons for exclusion of studies at full-text stage are reported in the PRISMA flow diagram.
Study characteristics	17	Cite each included study and present its characteristics.	Page 13-14 Characteristics of included studies are presented in Table 1.
Risk of bias in studies	18	Present assessments of risk of bias for each included study.	Page 13 Risk of bias was assessed using the Cochrane RoB 2 tool (Section 2.4)
Results of individual studies	19	For all outcomes, present, for each study: (a) summary statistics for each group (where appropriate) and (b) an effect estimate and its precision (e.g. confidence/credible interval), ideally using structured tables or plots.	Page 13-14 Results of individual studies are presented in Table 1 and described in the Results section.
Results of syntheses	20a	For each synthesis, briefly summarise the characteristics and risk of bias among contributing studies.	Page 13 Summary of included studies

Section and Topic	Item #	Checklist item	Location where item is reported
			and their characteristics are described in Section 3.2 Study Characteristics.
	20b	Present results of all statistical syntheses conducted. If meta-analysis was done, present for each the summary estimate and its precision (e.g. confidence/credible interval) and measures of statistical heterogeneity. If comparing groups, describe the direction of the effect.	Page 13-14 Results of meta-analyses (including relapse rates, MRI outcomes, and hepatotoxicity) are presented in Section 3.3 and Figures 2.1–2.3.
	20c	Present results of all investigations of possible causes of heterogeneity among study results.	NA Subgroup analyses were not performed.
	20d	Present results of all sensitivity analyses conducted to assess the robustness of the synthesized results.	NA Sensitivity analyses were not performed.
Reporting biases	21	Present assessments of risk of bias due to missing results (arising from reporting biases) for each synthesis assessed.	NA Reporting bias was not formally assessed due to the limited number of included studies.
Certainty of evidence	22	Present assessments of certainty (or confidence) in the body of evidence for each outcome assessed.	NA Certainty of evidence was not assessed using GRADE.
**DISCUSSION**			
Discussion	23a	Provide a general interpretation of the results in the context of other evidence.	Page 15 General interpretation of results is provided in Section 4.0 Discussion.
	23b	Discuss any limitations of the evidence included in the review.	Page 17 Limitations of the included evidence are discussed in Section 4.1 Limitations.
	23c	Discuss any limitations of the review processes used.	Page 17 Limitations of the review process

Section and Topic	Item #	Checklist item	Location where item is reported
			are discussed in Section 4.1 Limitations.
	23d	Discuss implications of the results for practice, policy, and future research.	Page 17 Implications for practice and future research are discussed in Sections 4.2 Clinical Implications and 4.3 Future Directions.
**OTHER INFORMATION**			
Registration and protocol	24a	Provide registration information for the review, including register name and registration number, or state that the review was not registered.	PROSPERO registration (ID: 1323474) reported in Methods section.
	24b	Indicate where the review protocol can be accessed, or state that a protocol was not prepared.	Protocol not publicly available.
	24c	Describe and explain any amendments to information provided at registration or in the protocol.	No amendments to protocol reported.
Support	25	Describe sources of financial or non-financial support for the review, and the role of the funders or sponsors in the review.	No funding information reported in the manuscript.
Competing interests	26	Declare any competing interests of review authors.	Conflict of Interest: The authors declare no conflict of interest.
Availability of data, code and other materials	27	Report which of the following are publicly available and where they can be found: template data collection forms; data extracted from included studies; data used for all analyses; analytic code; any other materials used in the review.	Data availability statement included: data available upon reasonable request.

Moher, D.; Liberati, A.; Tetzlaff, J.; Altman, D.G. Preferred Reporting Items for Systematic Reviews and Meta-Analyses: The PRISMA Statement. *PLOS Med.* **2009**, *6*, e1000097–269, https://doi.org/10.1371/journal.pmed.1000097.
